# The application value of contrast-enhanced computed tomography in cervical lymph node metastasis of oral carcinomas: A protocol for systematic review and meta-analysis

**DOI:** 10.1097/MD.0000000000027654

**Published:** 2021-11-19

**Authors:** Hao Zhang, Shou-Kang Yang, Xiao-Wen Jiang, Guo-Lei Deng, Kun-Qin Li, Yu-Fang Long

**Affiliations:** a Department of Oral and Maxillofacial Surgery, The First People's Hospital of Chenzhou, Chenzhou, Hunan, China; b Department of Stomatology, The First People's Hospital of Chenzhou, Chenzhou, Hunan, China; c Department of Radiology, The First People's Hospital of Chenzhou, Chenzhou, Hunan, China.

**Keywords:** cervical lymph node metastasis, contrast-enhanced computed tomography, oral carcinomas, oral cavity cancer, squamous cell carcinoma

## Abstract

**Background:**

Oral carcinomas is a concerning condition around the world. Globally, it is the 11th most common form of cancer. Over 90% of oral carcinomas are squamous cell carcinomas. Smoking, tobacco intake, smokeless tobacco (snuff or chewing tobacco), excessive sunlight exposure, alcohol, betel nut consumption, human papillomavirus, and reverse end smoking are the most common causes of oral carcinomas. Oral carcinomas are prone to neck lymph mode metastasis, which has an impact on the prognosis of patients and the five-year survival rate. Thus, precise lymph node metastasis and staging of oral carcinomas are critical. With the development of nuclear medicine and molecular imaging, an increasing number of studies have found that Contrast-Enhanced Computed Tomography (CECT) has high diagnostic value for tumors.

**Methods and analyses:**

The reviewers will conduct a thorough search for related literature in 6 online databases, including The Cochrane Library, PubMed, WanFang database, Web of Science, Chinese biomedical literature database, and the China National Knowledge Infrastructure. The authors will obtain full text of studies deemed to be eligible to extract and synthesize data. The present systematic review will be reported in accordance with the Preferred Reporting Project (PRISMA-P) of the 2015 System Review and Meta-Analysis Protocol.

**Results:**

The present systematic analysis will pool the results of individual studies to assess the value of CECT in cervical lymph node metastasis of oral carcinomas.

**Conclusion:**

The results in the proposed research will determine whether CECT is an efficient diagnostic method for cervical lymph node metastasis of oral carcinomas.

**Ethics and dissemination:**

This study will utilize secondary data from pre-published studies. Therefore, an ethical clearance is not required. The research outcomes shall be disseminated in conference reports and peer-reviewed publications.

**OSF registration number:**

Oct 13, 2021.osf.io/k5nr9. (https://osf.io/k5nr9/).

## 1 Introduction

Oral carcinomas are a type of head and neck cancer, which has developed and progressed at an alarming rate around the world. Over 90% of oral carcinomas are pathological types of oral squamous cell carcinoma,^[1]^ representing the frequently diagnosed types of oral carcinomas.^[2]^ Nearly 50%of oral carcinomas fatalities are attributed to smoking.^[3]^ Oral carcinomas are prone to neck lymph mode metastasis, which affects the prognosis of patients and the five-year survival rate. It is still challenging to detect cervical lymph node metastasis from squamous cell carcinoma of the oral cavity at an early stage. Thus, preoperative staging is critical when treating oral carcinomas patients. Therefore, accurate lymph node metastasis and staging of oral carcinomas are crucial. The current clinical imaging methods to diagnose Oral carcinomas primarily include ultrasound, CT, MRI, etc. Each method has advantages and disadvantages. With the development of nuclear medicine and molecular imaging, an increasing number of studies have found that Contrast-Enhanced Computed Tomography (CECT) has high diagnostic value for tumors. However, no previous study has evaluated the diagnostic efficacy of cervical lymph node metastasis for oral carcinomas. Oral carcinomas neck metastatic lymph nodes are based on surgical pathology or pathological puncture as the gold standard. Clinically, MRI and CECT are often adopted as the first line of treatment for oral carcinomas. CECT also has some limitations, such as contrast agent allergy, contrast agent nephrotoxicity, and radiation exposure.^[4]^ Researchers have increasingly questioned CECT's general diagnostic accuracy. However, it is difficult to diagnose neck metastatic lymph nodes of oral carcinomas using MRI and CECT. The limitation of CT is that it scatters metal inlays, such as metal dentures, affecting the imaging diagnosis.^[5]^ Reportedly, 59% of oral carcinomas patients have dental artifacts when undergoing CT or MRI examinations.^[6]^

This article systematically reviews and analyses the research literature of CECT in diagnosing cervical lymph node metastasis in oral carcinomas and provides a reference for clinical practice to objectively evaluate the diagnostic value of CECT in diagnosing lymph node metastasis in oral carcinomas.

## 2 Aim

The present systematic review and meta-analysis aims to evaluate the applicational value of CECT to diagnose cervical lymph node metastasis of oral carcinomas in people aged over 18 years old as published in studies until July 19, 2021.

The proposed meta-analysis will analyse the sensitivity and specificity of diagnosing cervical lymph node metastasis in oral carcinomas. Accordingly, assessment outcomes will help clinicians make personalized preoperative assessment and treatment plan formulation for oral carcinomas patients and can be used as a critical pre-operative assessment method for oral carcinomas patients.

### 2.1 Study design

The present protocol has been registered online on OSF, the International prospective register of systematic reviews (https://osf.io/gmb4f/. OSF registration number: October 13, 2021.osf.io/k5nr9.). The study will adhere to the Preferred Reporting Items for Systematic reviews and Meta-Analyses 22 guidelines for reporting systematic reviews.

### 2.2 Criteria for considering studies for review

#### 2.2.1 Types of studies

The proposed systematic review and meta-analysis will consider all diagnostic studies and other articles related to CECT of cervical lymph node metastasis and staging of oral carcinomas.

### 2.3 Studies inclusion criteria

1. Literature type: Research literature on using CECT to diagnose cervical lymph node metastasis and staging of oral carcinomas.
2. Research type: diagnostic research and the number of cases will be greater than 10.
3. Research objects: all patients with oral carcinomas that have a clear diagnosis by surgery or pathology will be included.
4. Diagnostic laboratory methods: CECT diagnosis of cervical lymph nodes in oral carcinomas patients VS pathological diagnosis (Gold Standard).

### 2.4 Studies exclusion criteria

1. Articles with incomplete or incorrect data.
2. Animal experiments, meeting minutes, case reports, literature reviews.
3. When it is not possible to extract literature that assess the sensitivity and specificity of CT in diagnosing cervical lymph nodes in oral carcinomas patients.

### 2.5 Assessment of the quality of and risk of bias in included studies

Firstly, the reviewers will utilize EndNote X8 to screen and remove duplicate studies. Afterwards, 2 independent researchers will screen the documents according to the unified classification standards, and collectively discuss the differences encountered. The extracted data will include research author, publication time, enrolment, number of cases, diagnostic gold standard, true positive, false positive, true negative, false negative, etc. The quality of the included studies will be assessed. The assessment will consider internal and external validity and bias risk using the validated quality appraisal tool introduced by Hoy et al.^[7]^

## 3 Data synthesis and analysis

Stata 13.0 and RevMan 5.3 software will be used to analyse the extracted four-grid table data, calculate the combined sensitivity, combined specificity, combined positive likelihood ratio (1ikelihood ratio, +LR), combined negative likelihood ratio (negative Likelihood ratio, -LR), diagnostic odds ratio, and receiver operating characteristic curve (SROC curve). We will adopt *X*^*2*^ and *I*^*2*^ tests to assess the heterogeneity. In the case of heterogeneity, the source of the heterogeneity will be examined. If the heterogeneity is less than moderate, the fixed effects model is used. Meanwhile, if a high degree of heterogeneity is observed, the random effects model will be employed. Lastly, we will use the funnel chart to determine the size of the publication bias.

## 4 Assessment of publication bias

The authors will evaluate the presence of publication bias via a funnel chart, accompanied by standard statistical tests using Begg test and Egger test^[8,9]^ to determine publication bias. Moreover, the pruning and filling methods of Duval and Tweedie will be applied to examine the robustness of the research results against publication bias.^[10]^

## Author contributions

**Conceptualization:** Hao Zhang, Kun-Qin Li, Yu-Fang Long.

**Data curation:** Hao Zhang, Shou-Kang Yang, Xiao-Wen Jiang, Guo-Lei Deng, Kun-Qin Li, Yu-Fang Long.

**Formal analysis:** Hao Zhang, Shou-Kang Yang, Xiao-Wen Jiang, Kun-Qin Li, Yu-Fang Long.

**Funding acquisition:** Hao Zhang, Shou-Kang Yang, Xiao-Wen Jiang, Yu-Fang Long.

**Investigation:** Hao Zhang, Guo-Lei Deng.

**Methodology:** Yu-Fang Long.

**Project administration:** Shou-Kang Yang, Xiao-Wen Jiang, Guo-Lei Deng, Yu-Fang Long.

**Resources:** Hao Zhang, Xiao-Wen Jiang, Kun-Qin Li.

**Software:** Shou-Kang Yang, Guo-Lei Deng.

**Supervision:** Shou-Kang Yang, Xiao-Wen Jiang, Yu-Fang Long.

**Validation:** Hao Zhang, Kun-Qin Li, Yu-Fang Long.

**Writing – original draft:** Hao Zhang.

**Writing – review & editing:** Hao Zhang, Yu-Fang Long.
